# The healing potential of an acutely repaired ACL: a sequential MRI study

**DOI:** 10.1186/s10195-020-00553-9

**Published:** 2020-08-31

**Authors:** Andrea Ferretti, Edoardo Monaco, Alessandro Annibaldi, Alessandro Carrozzo, Mattia Bruschi, Giuseppe Argento, Gregory S. DiFelice

**Affiliations:** 1 Orthopaedic Unit and Kirk Kilgour Sports Injury Centre, S. Andrea Hospital, University of Rome La Sapienza, Rome, Italy; 2 Department of Radiology, S. Andrea Hospital, University of Rome La Sapienza, Rome, Italy; 3 Hospital for Special Surgery/Weill Cornell Medical Center, New York, NY USA

**Keywords:** Anterior cruciate ligament, Primary ACL repair, MRI, Knee

## Abstract

**Background:**

Recently, there has been renewed interest in primary anterior cruciate ligament (ACL) repair. The aim of this study is to report early clinical and radiological results of a consecutive series of acute ACL tears treated with arthroscopic primary ACL repair within 14 days from injury.

**Patients and methods:**

A consecutive series of patients with acute ACL tears were prospectively included in the study. Based on MRI appearance, ACL tears were classified into five types, and tissue quality was graded as good, fair, and poor. Patients with type I, II, and III tears and at least 50% of ACL tibial remnant intact with good tissue quality were ultimately included. Clinical outcomes were measured using the Tegner Lysholm Knee Scoring Scale (TLKSS), the Knee Injury and Osteoarthritis Outcome Score (KOOS), subjective and objective International Knee Documentation Committee (IKDC) scores, and KT-1000. Patients were also followed up with MRI evaluations at 1, 3, and 6 months postoperatively. ACL appearance was graded based on morphology (normal or abnormal) and signal intensity (isointense, intermediate, and hyperintense).

**Results:**

The mean TLKSS was 98.1, the mean subjective IKDC was 97.6, and the mean KOOS was 98.2. The objective IKDC score was A in eight of ten patients and B in two patients. KT-1000 measurements showed a maximum manual side-to-side difference of less than 2 mm in eight of ten patients, whereas two patients showed a difference of 3 mm. The morphology of the repaired ACL was normal (grade 1) at 1 month follow-up in ten of ten cases, and this appearance persisted at 3 and 6 months postoperatively. The signal intensity at 1 month postoperatively was graded as isointense (grade 1) in four of ten patients, intermediate (grade 2) in five of ten patients, and hyperintense (grade 3) in one of ten patients. At both 3 and 6 months postoperatively, the signal intensity was graded as isointense (grade 1) in nine of ten patients and intermediate (grade 2) in one of ten patients.

**Conclusions:**

Arthroscopic primary ACL repair performed acutely in a carefully selected group of patients with proximal ACL tears and good tissue quality showed good early clinical and radiological results.

**Level of evidence:**

Level 4.

## Introduction

Anterior cruciate ligaments (ACL) tears represent one of the most frequent orthopedics and sports medicine injuries in the athletically active population and often result in knee instability, functional impairment, and cartilage and meniscal diseases that eventually contribute to the development of posttraumatic arthritis [1, 2].

Open primary repair of acute ACL injuries (< 2 weeks from injury) was the standard treatment for ACL injuries until the middle to the late 1980s. Clinical results of these ACL repairs were initially promising, but midterm follow-up studies revealed much higher rates of instability and pain, and eventually, ACL reconstruction became the gold standard technique [3–6]. It is likely that inaccurate patient selection that treated all tear types in the same way, higher morbidity surgical techniques performed through open arthrotomies, and old-fashioned rehabilitation protocols focused on early cast immobilization negatively affected the outcomes of ACL repair in this early era of ACL surgery [6–8].

Recently, taking advantage of modern arthroscopic surgical techniques, materials, and devices, there has been a renewed interest in primary repair of ACL. In the last several years, some studies on ACL repair have shown good to excellent results with a failure rate ranging from 7% to 15% of cases [9–13]. Most articles recommend surgery within 4 weeks from injury; however, there is a wide variety of delay until treatment, including at least one case report of a patient treated 11 years after injury [14]. Since the renewal of interest in ACL primary repair has only been recent, there has been little investigation regarding MRI evaluation after ACL primary repair [7, 16, 17].

Therefore, the aim of this study is to report early clinical and radiological results of a consecutive series of acute ACL tears that underwent arthroscopic primary ACL repair within 14 days from injury. Our hypothesis is that early selective primary ACL repair performed in patients with proximal tears and good tissue quality gives satisfactory results, including good clinical and stability outcomes. In addition, that sequential MRI evaluation will reveal a good grade of healing and maturation of the repaired ACL tissues.

## Patients and methods

The study was approved by our Institutional Review Board. All patients referred to our hospital from January to June 2019 with an acute ACL tear who were operated on within 2 weeks of their injury were provisionally selected for this prospective study. All patients were evaluated with a careful history and physical examination (Lachman and pivot shift test) followed by preoperative evaluation with a 1.5-T MRI to confirm diagnosis. Exclusion criteria were: partial ACL tears, multiligamentous injury, and previous ipsilateral severe injury or operation, in addition to patients who were unwilling to participate in the study. Based on their MRI appearance, ACL tears were graded according to the classification of Van der List et al. into five types [11]. Type I is a proximal avulsion of the ligament from its femoral insertion (distal remnant length > 90% total ligament length), type II is a proximal tear (75–90%), type III is a midsubstance tear (25–75%), type IV is a distal tear (10–25%), and type V is a distal avulsion tear (0–10%) [11]. Moreover, the tissue quality of the ACL on the preoperative MRI was evaluated to predict eligibility for ACL repair and classified as good, fair, and poor according to van der List and Di Felice [15]. Patients with type I–III tears with at least 50% of tibial ACL remnant intact along with good tissue quality were ultimately prospectively selected for the study. Demographic data including age, gender, body mass index (BMI), delay from injury to surgery, mechanism of injury, and concomitant tears were collected. In the operating room, prior to surgical intervention, an examination under anesthesia was performed to confirm the preoperative diagnosis and to more accurately quantify the laxity with the Lachman and pivot shift tests. Intraoperatively, the ACL was assessed by direct visualization and by probing during initial diagnostic arthroscopy to confirm the tear type and tissue quality. Patients with tear type III with less than 50% of tibial ACL remnant intact, types IV and V tears, and those with fair or poor tissue quality were excluded, and the operation was switched to an ACL reconstruction with autograft hamstrings. Patients were also excluded if a severe cartilage tear was found (Outerbridge grade III or IV) at arthroscopy. Patients with tear types I–III with good ACL remnant tissue quality (as defined by the presence of a tibial stump at least 50% in length with only mild interstitial tearing and the ability to hold sutures) at arthroscopy underwent primary repair of the ACL, whereas those with inadequate tissue, as mentioned above, were converted to ACL reconstruction. All patients gave their preoperative consent to undergo either ACL repair or ACL reconstruction based on the above criteria.

Clinical outcomes were measured at final follow-up using the Tegner Lysholm Knee Scoring Scale (TLKSS), the Knee Injury and Osteoarthritis Outcome Score (KOOS), and both subjective and objective International Knee Documentation Committee (IKDC) scores. Clinical assessment via Lachman test, pivot shift test, and KT-1000 arthrometer (MedDmetric, San Diego, CA) measurements were performed at the 6-month visit. Lachman and pivot shift tests were graded according to the IKDC. Lachman manual test was graded as A (normal) in the presence of 1–2 mm anterior translation and a firm endpoint, B (nearly normal) with 3–5 mm anterior translation, C (abnormal) with 5–10 mm anterior translation with a soft endpoint, and D (severely abnormal) with greater than 10 mm anterior translation. Pivot shift test was graded as 0 (normal), 1 (glide), 2 (clunk), and 3 (locked subluxation). Patients were also followed up with MRI evaluations at 1, 3, and 6 months postoperatively.

### Surgical technique

**Fig. 1:**
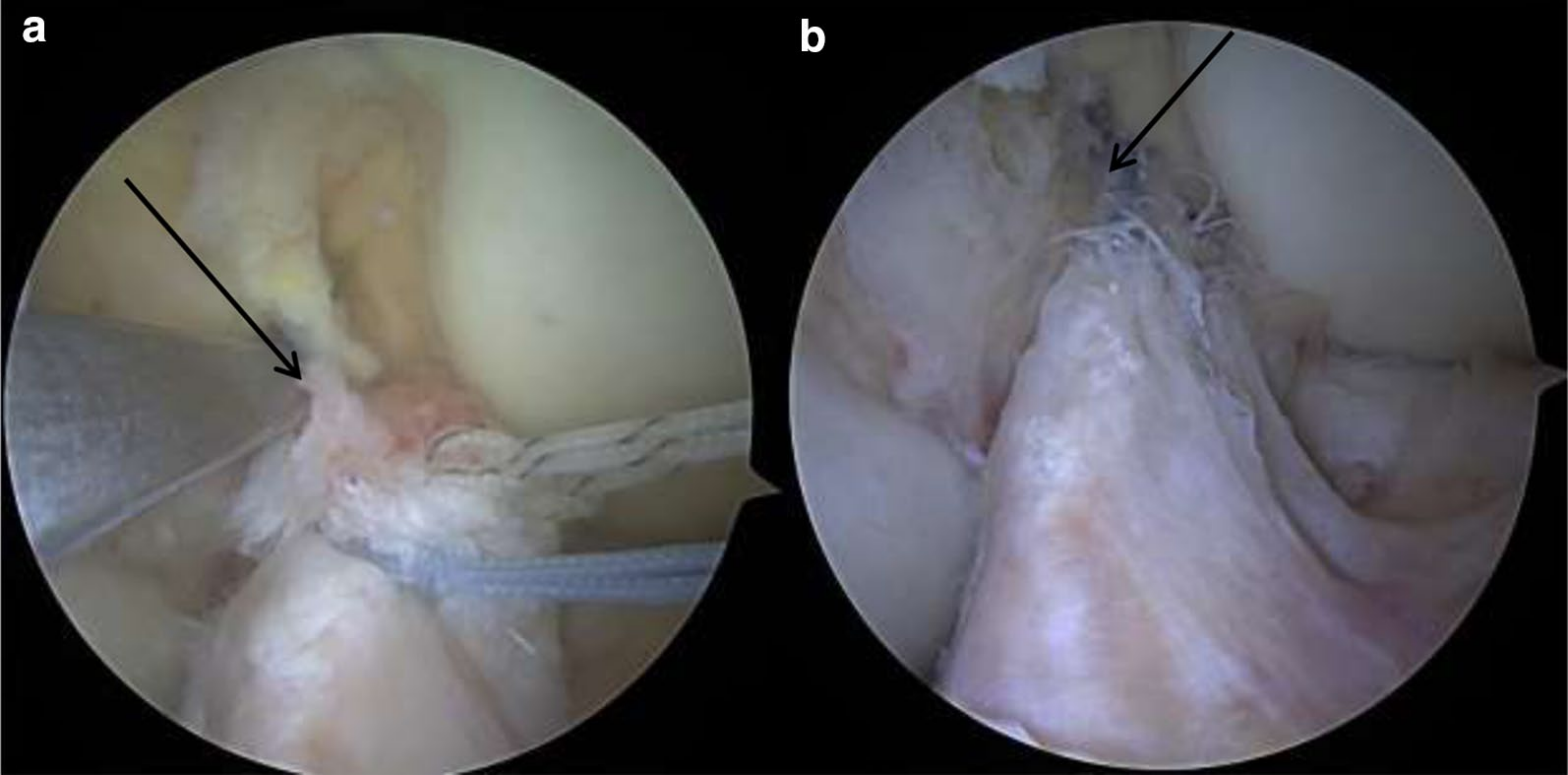
Repair of a type III ACL tear. **a** The tibial stump was prepared with no. 2 FiberWire^®^ (blue) and TigerWire^®^ (striped). Femoral guide placed at the level of the femoral stump (black arrow). **b** Final arthroscopic image of a complete primary ACL repair. Black arrow indicates anatomic reapproximation of the stumps

All surgeries were performed by the senior author (A.F.). The patient was placed in supine position, and the operative leg was prepared and draped for a standard knee procedure. Knee reached at least 90° degrees of flexion. A transtendinous portal was used for diagnostic arthroscopy, and the anteromedial portal was used as a working portal. The torn ACL was carefully evaluated and probed to identify the tear type and determine tissue quality. For those knees with type I–III tears with over 50% of tibial remnant intact and good tissue quality, we proceeded with the ACL repair technique. An accessory anterolateral portal was created, and a 6-mm PassPort cannula (Arthrex) was inserted to facilitate suture passage and management. The ACL remnant on the tibial side was prepared by suture passage into the ligament with a scorpion suture passer using no. 2 FiberWire^®^ and TigerWire^®^ stitches (Arthrex) that were looped through the ligament using a lasso-loop knot-tying configuration (Fig. 1a) [16]. The stitches were passed through the anteromedial and posterolateral bundle of the ACL. The strength of suture fixation was tested by pulling traction on the ends of the stitches. Then, a femoral outside–in ACL guide was used to create a femoral tunnel. The guide was placed at the origin of the femoral stump for anatomic guidance. Note that the femoral stump was not debrided at all to ensure anatomic positioning. The femoral tunnel was drilled using an outside–in technique with a 3.5-mm drill. A FiberStick™ no. 2 (Arthrex) was then passed from outside to in through the guide trocar and retrieved with a grasper from the anteromedial portal. The FiberStick™ was then used to pass the repair stitches up through the femoral tunnel to reapproximate the tibial ACL remnant to the femoral ACL stump. The repair stitches were then passed through a Dog Bone™ Button (Arthrex). After cycling the knee, the repair stitches were tensioned with the knee in full extension and tied off with alternating half hitches. Finally, the repaired ACL was probed and evaluated at different degrees of flexion to confirm the integrity of the repair.

### Postoperative rehabilitation protocol

A short-ROM knee brace was applied postoperatively for the first 6 weeks. The brace was locked in extension for the first week, and then unlocked for the remaining 5 weeks. Weight bearing with brace and crutches was allowed as tolerated on postoperative day 1. The first week was focused on pain and swelling control with ice and antiinflammatory drugs. Range of motion exercises were started 1 week after surgery with the goal to achieve and maintain full extension and progressively recover flexion. Full ROM was usually obtained by a maximum of 6 weeks after surgery. The brace was removed at 6 weeks after surgery, and patients started a supervised strengthening program. Sports activities were allowed 6 months postoperatively.

### Post-op MRI evaluation

Patients were assessed with a 1.5-T MRI preoperatively and then sequentially at 1, 3, and 6 months after surgery. An experienced musculoskeletal radiologist was asked to describe the imaging. The radiologist was unblinded about the type of surgical procedure but blinded about the postoperative timing of the MRI. The ACL appearance was classified based on morphology and signal intensity. Morphology was graded as normal (grade 1) or abnormal (grade 2). Signal intensity was graded as compared with the posterior cruciate ligament (PCL) signal intensity: grade 1 (isointense), grade 2 (intermediate intensity), or grade 3 (hyperintense) [13].

## Results

### Demographic data

During the study period, a total of 15 patients were admitted at our department with acute ACL tears. Of these patients, five were excluded (two refused ACL repair and three were judged irreparable), and they subsequently underwent ACL reconstruction with a hamstring graft. This left a total of ten patients who were treated with arthroscopic primary ACL repair and composed the study group. Patient demographic data, BMI, mechanism of injury, delay from injury to surgery, concomitant tears, MRI, and arthroscopic tear types are presented in Table 1. There were five males and five females. The mean age at surgery was 31 years (range 18–46 years), the mean BMI was 22.15 kg/m^2^, and the mean delay from injury to surgery was 7 days (range 3–12 days). All injuries were sports related (football, skiing, rugby, boxing, and volleyball). Two patients had concomitant tears: one bucket handle tear of medial meniscus treated with partial meniscectomy and one Segond fracture treated with suture anchor repair. Among the repaired cases, two were type I with good tissue quality, five cases were type II (four good and one fair tissue quality), and three cases were type III with at least 50% of tibial ACL remnant and good tissue quality.

**Table 1:** Demographic data

	Age, years	Sex	BMI, kg/m^2^	Days injury–surgery	Mechanism of injury	Other tears	MRI type and tissue quality	Arthroscopic type and tissue quality
Patient 1	18	Female	19.29	3	Volley		Type II (good quality)	Type II (good quality)
Patient 2	24	Female	19.95	11	Skiing	Segond fracture	Type II (good quality)	Type I (good quality)
Patient 3	31	Female	20.58	5	Skiing		Type III (good quality)	Type III (good quality)
Patient 4	46	Female	21.16	12	Skiing		Type II (good quality)	Type II (good quality)
Patient 5	22	Male	24.41	7	Volley		Type III (good quality)	Type II (good quality)
Patient 6	42	Female	21.71	6	Rugby		Type II (good quality)	Type I (good quality)
Patient 7	30	Male	24.05	8	Football		Type II (good quality)	Type II (good quality)
Patient 8	27	Male	23.32	5	Football		Type III (good quality)	Type III (good quality)
Patient 9	41	Male	24.49	4	Football	Bucket handle medial meniscus	Type III (good quality)	Type III (good quality)
Patient 10	29	Male	22.54	5	Boxing		Type II (good quality)	Type II (fair quality)
Mean ± SD	37 ± 9.22		22.15 ± 1.9	6.6 ± 2.95				

*SD* standard deviation, *BMI* body mass index

### Clinical and radiological assessment

Patients had routine office follow-up visits at 1 week, 2 weeks, and then at 1, 3, and 6 months after surgery. Clinical outcomes were collected at final follow-up and are presented in Table 2. The mean TLKSS score was 98.1, the mean subjective IKDC score was 97.6, and the mean KOOS score was 98.2. The objective IKDC score was A in eight of ten patients, and B in two patients. Lachman test was A (normal) in eight of ten patients and B (nearly normal) with a firm endpoint in two patients. Pivot shift test was 0 (normal) in nine of ten patients and one(glide) in one patient. All patients achieved full ROM. KT-1000 measurements showed a maximum manual side-to-side difference of less than 2 mm in eight of ten patients, whereas two patients showed a difference of 3 mm (Table 2). No specific complications were recorded in this series of patients, and neither revision nor reoperation surgery was required at final follow-up.

**Table 2:** Clinical outcomes

	Lysholm score	KOOS	Subjective IKDC	Objective IKDC	Difference on KT-1000 maximum manual testing at 30° (mm)	Lachman test	Pivot shift test
Patient 1	100	100	100	A	1	A (normal)	0 (normal)
Patient 2	93	96.4	97.7	A	0	A (normal)	0 (normal)
Patient 3	100	100	100	A	2	A (normal)	0 (normal)
Patient 4	100	98.2	100	A	1	A (normal)	1 (glide)
Patient 5	100	99.4	100	A	2	A (normal)	0 (normal)
Patient 6	98	99.4	98.9	A	1	A (normal)	0 (normal)
Patient 7	94	95.2	92	B	3	B (nearly normal)	0 (normal)
Patient 8	100	98.8	95.4	B	3	B (nearly normal)	0 (normal)
Patient 9	98	97.6	93.1	A	1	A (normal)	0 (normal)
Patient 10	98	97	98.9	A	0	A (normal)	0 (normal)
Mean ± SD	98.1 ± 2.6	98.2 ± 1.6	97.6 ± 3				

*SD* standard deviation, *KOOS* Knee injury and Osteoarthritis Knee Outcome Score, *IKDC* International Knee Documentation Committee

**Fig. 2:**
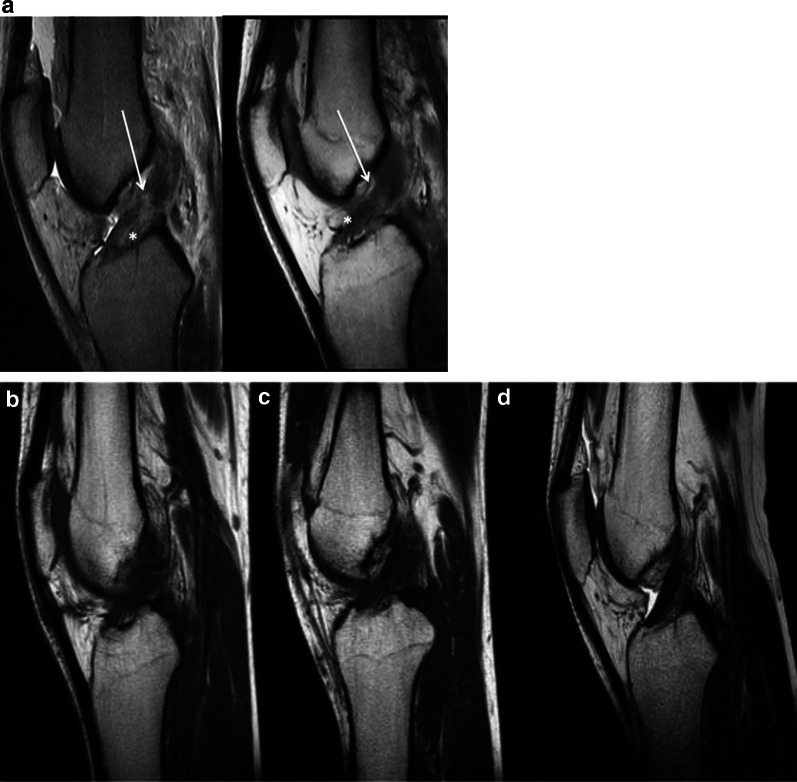
Pre- and postoperative images of patient #5. **a** Sagittal preoperative MRI T2-TSE (left) and T1-TSE (right). Tear location (arrow): type III; tissue quality (asterisk): good **b** Sagittal T1-TSE 1 month post-op. Morphology: grade 1 (normal); signal intensity: grade 2 (intermediate) **c** Sagittal T1-TSE 3 months post-op. Morphology: grade 1 (normal); signal intensity: grade 1 (isointense) **d** Sagittal T1-TSE 6 months post-op. Morphology: grade 1 (normal); signal intensity: grade 1 (isointense) *TSE* turbo spin-echo

**Fig. 3:**
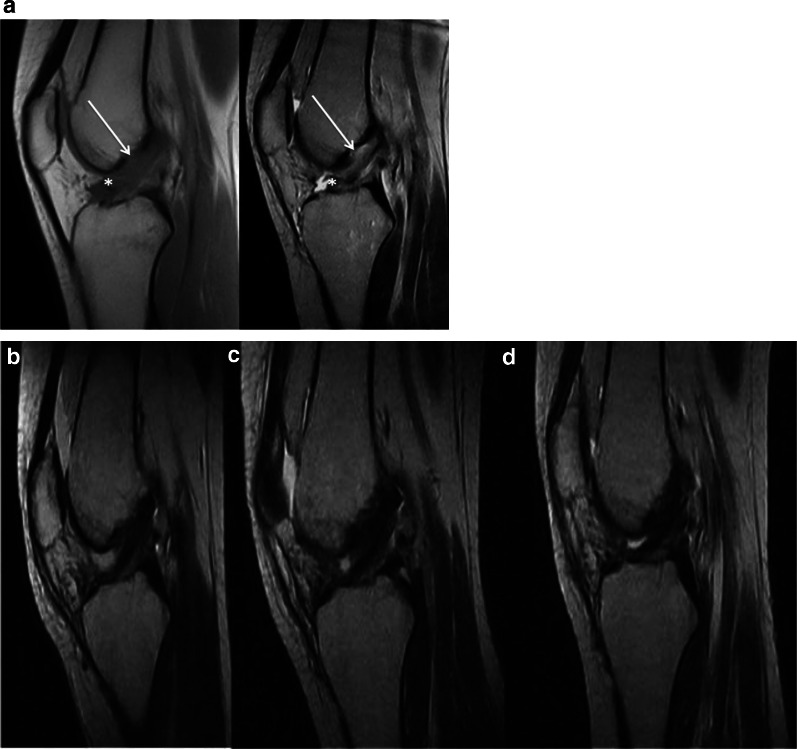
Pre- and postoperative images of patient #3. **a** Sagittal preoperative MRI T1-TSE (left) and T2-TSE (right). Tear location (arrow): type III; tissue quality (asterisk): good **b** Sagittal T2-TSE 1 month post-op. Morphology: grade 1 (normal); signal intensity: grade 1 (isointense) **c** Sagittal T2-TSE 3 months post-op. Morphology: grade 1 (normal); signal intensity: grade 1 (isointense)  **d** Sagittal T2-TSE 6 months post-op. Morphology: grade 1 (normal); signal intensity: grade 1 (isointense) *TSE* turbo spin-echo

**Fig. 4:**
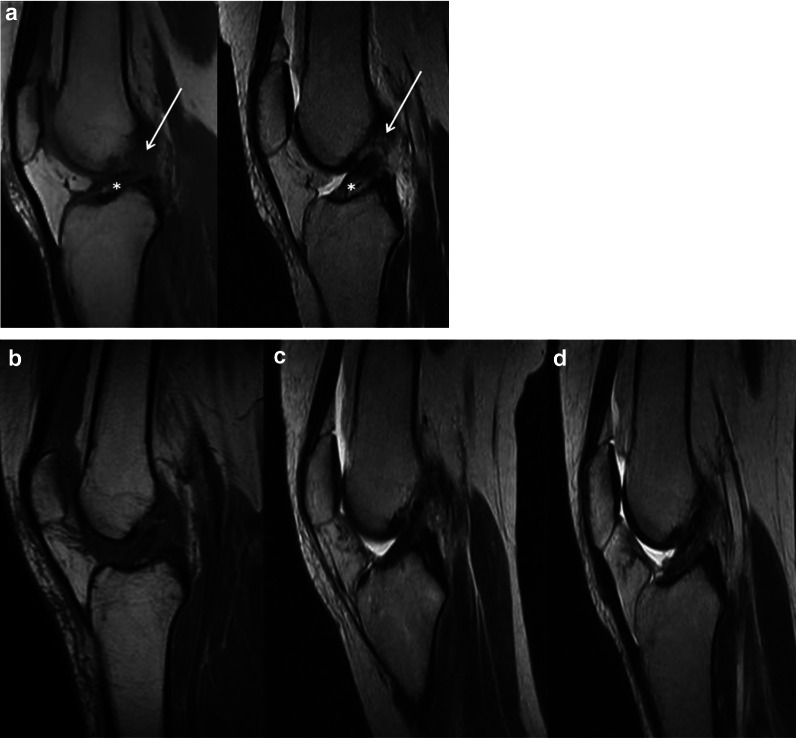
Pre- and postoperative images of patient #6. **a** Sagittal preoperative MRI T1-TSE (left) and T2-TSE (right). Tear location (arrow): type II; tissue quality (asterisk): good **b** Sagittal T2-TSE 1 month post-op. Morphology: grade 1 (normal); signal intensity: grade 1 (isointense) **c** Sagittal T2-TSE 3 months post-op. Morphology: grade 1 (normal); signal intensity: grade 1 (isointense) **d** Sagittal T2-TSE 6 months post-op. Morphology: grade 1 (normal); signal intensity: grade 1 (isointense) *TSE* turbo spin-echo

Postoperative MRIs were performed in all cases at 1, 3, and 6 months after ACL repair. The morphology of the repaired ACL was normal (grade 1) at 1 month follow-up in ten of ten cases (100%), and this appearance persisted at 3 and 6 months postoperatively. Regarding signal intensity, four of ten patients showed an isointense signal (grade 1) at 1 month postoperatively, five of ten patients showed an intermediate intensity (grade 2), and one showed a hyperintense signal (grade 3). At both 3 and 6 months postoperatively, the signal intensity was graded as isointense (grade 1) in nine of ten patients and intermediate (grade 2) in one of ten patients. The MRI images of three patients are reported in Figs. 2, 3, and 4.

## Discussion

The main finding of this study is that MRI appearance of an acutely repaired ACL seems normal or close to normal in all but one case by 3 months and normal in all cases by 6 months. These results seem to provide evidence suggestive that an acutely torn ACL, whose stumps can be acutely reapproximated in tension, has the potential to heal. Another important finding is that all patients showed acceptable clinical results with an IKDC objective score of grade A in eight of ten patients and grade B in only two of ten. No failures (IKDC grades C or D) were recorded in this preliminary series of patients. Furthermore, arthrometric evaluation using KT-1000 showed a side-to-side difference of less than 2 mm on maximum manual testing in eight of ten patients and 3 mm in two of ten patients.

Magnetic resonance imaging is a valuable tool that allows the anatomical integrity of the ACL to be assessed by visualizing the entire anatomy from femoral origin to tibial insertion [17]. Multiple previous studies have evaluated the role of postoperative MRI after ACL reconstruction both after bone–patellar tendon bone (BPTB) and hamstrings grafts [14, 17]. However, the graft maturation process is a distinctly different biological situation than the healing of the native ACL that has been repaired, and thus it is felt that the relevance to primary ACL repair is limited. Several studies have evaluated the role of postoperative MRI on graft maturation after ACL reconstruction, however, only a few studies have assessed the role of MRI after ACL repair [7, 17, 18]. Several MRI studies have been done in animal ACL repair models by the group led by Dr. Murray. They found that MRI had an important role in predicting the size and mechanical properties of the healing ACL in a large animal model. They also used MRI to show that the cross-sectional area of the ligament and its signal intensity, which indicate the quantity and the quality of the tissue respectively, can be combined to approximate the maximum strength of the repaired ACL [19, 20].

The first study in humans that assessed the role of postoperative MRI after ACL primary repair was conducted by Di Felice et al. They analyzed 37 MRIs from 36 patients with a mean surgery–MRI interval of 1.5 years and showed that postoperative MRI after ACL primary repair is useful to identify rerupture of the repaired ACL. In addition, they reported that the ACL appears hyperintense in the first year after surgery as compared with the intensity of the PCL, although it becomes isointense by 2 years after surgery [7]. These findings suggest that the ACL is healing and remodeling in the ensuing years after surgery.

In contrast to this timing, other authors documented MRI changes in a repaired ACL and reported a gradual decrease in signal intensity between 3 and 6 months after internal brace ligament augmentation (IBLA) and dynamic intraligamentary stabilization (DIS), resulting in a nearly normal signal intensity within 1 year after procedure. Furthermore, the authors found that the time course of changes observed in the repaired ligament was different from that observed after standard ACL reconstruction, in which the signal intensity of the ACL graft gradually increases after surgery, peaks between 4 and 8 months, and decreases with graft maturation during the ligamentization process [21, 22].

Although it is difficult to directly compare signal intensity from different studies because the signal intensity changes depend on hardware, sequence parameters, and the normalization process selected, our data support previous studies evidencing that the healing process of a repaired ACL is different than that of a reconstruction in that this does not involve a “ligamentization” phase. Certainly, in this study and others, at least the morphology of the repaired ACL showed a normal or close to normal appearance within 6 months postoperatively in all cases [23].

It is clear that the ACL does have healing potential, and this has been studied by several authors. Murray et al. underlined that the healing of the ACL has differences compared with that of other dense connective tissues: the lack of any bridging scar between the stumps and the presence of an epiligamentous phase between 3 and 8 weeks after injury. The stumps retraction and the lack of healing of the ligament could be due to the presence of a layer of synovial cells, which through the expression on the surface of a particular actine isoform called the alpha smooth muscle actine, differentiate into myofibroblasts [24]. Our technique is based on an acute surgery performed in the inflammatory phase of the healing process aiming to maximize the potential of healing. The goal of the procedure is to reapproximate the tibial ACL remnant to the femoral stump, which is left in situ at the level of femoral ACL footprint, when they are still in the original length, avoiding any debridement in both sides which could damage the healing potential of the remnants or enlarge the gap. In any case, we feel that repair should be performed before formation of a large number of contractile myofibroblasts occurs and leads to retraction of the stumps, consequentially increasing the gap. The presented technique is mainly focused on assisting the body with its natural tendency to heal by approximating the torn tissues, preventing contraction of the tissues and widening of the gap.

In the present series there was no augmentation with suture nor scaffold due to the desire to optimize the favorable healing environment. However, some authors have suggested the use of an internal brace (IB) at the time of ACL repair to protect the biological environment and prevent displacement of the tissue in the healing phase by controlling anterior translation [13, 21, 25, 26]. Other authors, in cases of delayed surgery, have promoted the use of biological scaffold or stimulation, especially when a shorter retracted stump is found, to optimize the healing environment between the torn ends of the ligament [27].

It is felt that proximal tears of ACL have a better chance of healing when compared with midsubstance injury patterns. In their systematic review, van der List et al. showed that indeed, when the historic open ACL repair data were stratified by tear type, patients with proximal tears had better results than those with midsubstance injuries [10]. This is likely due to several factors including ACL vascularization. Arterial supply to the majority of ACLs arises from the middle genicular artery, while the distal end is vascularized by the inferior genicular artery. The blood supply to the ACL is not homogeneous: the proximal ACL receives a greater blood supply than the middle and the distal third. It is likely that this asymmetric vascularity may explain some of the scattered cases of spontaneous healing of proximal ACL tears [10, 28–30].

The concept of modern-day, selective, arthroscopic ACL primary repair is focused on the differential healing capacity of the ACL based upon its blood supply. It has been shown that excellent short- and medium-term results can be obtained by repairing a select group of proximal ACL tears with a dual suture anchor technique as long as there is an appropriate tear type with good to excellent tissue quality [10, 24]. Others have expanded on this work and developed other techniques; however, they are all specifically focused on the proximal tear pattern. The results of these techniques have been reported in several recent reviews and metaanalyses of literature on ACL repair with some conflicting results noted. The first, which was focused exclusively on arthroscopic ACL repair techniques, examined 13 studies and 1101 patients showing how three different techniques (primary repair with static augmentation, dynamic augmentation, and without augmentation) are safe, with failure rates between 7% and 11%. The second, which looked at 28 studies and 2401 patients, reported how ACL reconstruction results in better survivorship, and patients perceived postoperative improvement higher than that of the repair. However, note that this review included some long-term follow-up studies from the nonselective open repair days, likely limiting the applicability of their conclusions. Regardless, both studies stressed the obvious lack of long-term studies on the modern technique of arthroscopic ACL repair [9, 31]. It is felt that it is early in the resurgence of interest in ACL primary repair and that, as indications and techniques evolve, our understanding and outcomes will improve proportionately. From our findings, it does appear that there is certainly healing potential that can be harnessed by primary repair to avoid reconstruction in proximally torn ACLs.

Limitations are present in this study. First of all, the relatively low number of patients; we should consider that the very strict inclusion criteria result in a quite complicated selection of patients. Recent similar reports on this subject were only case reports and limited series. Another important limitation is that MRI scans were classified according to subjective criteria (morphology and intensity signal) and evaluated only once by a single experienced musculoskeletal radiologist with lack of any interobserver or interobserver variability. Moreover, the radiologist was unblinded about the surgical procedure. A third limitation is that this is a case series study, and no statistical analysis was applied. Finally, this is mainly a radiological study lacking a consistent 2-year minimum clinical follow-up.

## Conclusions

On the basis of this preliminary radiological study, an early anatomic repair of the ACL in mostly proximal tears seems to be able to maximize the potential of healing of the native ACL. MRI showed early recovery of a normal morphology, and signal intensity indicated a healing rather than remodeling process as is currently observed in autologous or allograft reconstructions. Despite the excellent MRI appearance of the repaired ACL since the first postoperative period, the actual ability of the repaired ligament to sustain the load of the native ligament cannot be ultimately assessed by a short-term imaging study. Further studies with a longer clinical follow-up are needed before the presented procedure can be recommended as a suitable and reproducible repair technique.

## Data Availability

The database generated during and/or analyzed during the current study is not publicly available but is available from the corresponding author on reasonable request.

## Abbreviations

ACL: Anterior cruciate ligament
TLKSS: Tegner Lysholm Knee Scoring Scale
MRI: Magnetic resonance imaging
PCL: Posterior cruciate ligament
IKDC: International Knee Documentation Committee
KOOS: Knee Injury and Osteoarthritis Outcome Score
ROM: Range of motion
BMI: Body mass index
SD: Standard deviation
BTB: Bone–patellar tendon bone
IBLA: Internal brace ligament augmentation
DIS: Dynamic intraligamentary stabilization
IB: Internal brace
